# Cost-effectiveness and budget impact analysis of lisdexamfetamine versus methylphenidate for patients under 18 with attention-deficit/hyperactivity disorder in Iran

**DOI:** 10.1186/s13034-023-00664-1

**Published:** 2023-10-10

**Authors:** Amirmohammad Tajik, Shekoufeh Nikfar, Sepideh Elyasi, Omid Rajabi, Mehdi Varmaghani

**Affiliations:** 1 School of Pharmacy, Mashhad University of Medical Sciences, Mashhad, Iran; 2 Department of Pharmacoeconomics and Pharmaceutical Administration, School of Pharmacy, Tehran University of Medical Sciences, Tehran, Iran; 3 Department of Clinical Pharmacy, School of Pharmacy, Mashhad University of Medical Sciences, Mashhad, Iran; 4 Department of Pharmaceutical Control, Faculty of Pharmacy, Mashhad University of Medical Sciences, Mashhad, Iran; 5 Social Determinants of Health Research Center, Mashhad University of Medical Sciences, Mashhad, Iran; 6 Department of Management Sciences and Health Economics, School of Health, Mashhad University of Medical Sciences, Mashhad, Iran

**Keywords:** Lisdexamfetamine dimesylate, Methylphenidate, Attention deficit disorder with hyperactivity, Cost-effectiveness analysis, Iran

## Abstract

**Background:**

Lisdexamfetamine (LDX) and Methylphenidate (MPH) are stimulant agents that have been shown to provide significant benefits in the management of attention-deficit/hyperactivity disorder (ADHD) in patients.

**Aim:**

This study aimed to assess the cost-effectiveness and the budget impact of LDX compared to MPH as the first-line treatment for ADHD.

**Methods:**

A one-year cost-effectiveness analysis (CEA) was conducted to compare the effects of LDX and MPH in reducing disease symptoms and patient costs and improving quality of life (QoL) from a social perspective. Clinical data were obtained using the EQ-5D questionnaire. In contrast, economic data were sourced from the official website of the Iranian Food and Drug Association (FDA), the national book of tariffs, and specific questionnaires designed to evaluate patients' direct and indirect costs. 197 patients were included in the study, including individuals who sought psychiatric evaluation at a hospital in Mashhad and those who obtained ADHD medications from governmental pharmacies. The cost-effectiveness of the study medicine was assessed using the decision tree method, and the results were presented as the Incremental Cost-Effectiveness Ratio (ICER). Deterministic Sensitivity Analysis (DSA) and Probabilistic Sensitivity Analysis (PSA) were performed to assess the robustness of the findings. Additionally, a Budget Impact Analysis (BIA) was conducted over five years, considering three different scenarios, to evaluate the financial implications of incorporating LDX into the national pharmaceutical system.

**Results:**

The ICER for LDX therapy compared to MPH was estimated at USD 264.28 (with an incremental cost of USD 54.9, incremental effectiveness of 0.208, and Quality-Adjusted Life Years (QALYs) gained of 0.765). The PSA indicated a 0.994% probability of LDX being cost-effective, considering a threshold of USD 2450 per QALY. Furthermore, the DSA revealed that the acquisition cost of LDX influenced the model's sensitivity. The BIA demonstrated that incorporating LDX into Iran's healthcare system would result in a financial burden of approximately $368,566 in the first year, representing an additional cost of $11,154 compared to the non-availability of this medicine and the use of previous medications. It is projected that by 2027, the financial burden of treating ADHD with LDX will reach approximately USD 443,879 over five years, amounting to an increase of $71,154 compared to the absence of this medicine.

**Conclusion:**

From a social perspective, the inclusion of LDX in the treatment regimen for ADHD is associated with higher costs and an increased financial burden. However, based on our analysis, LDX appears to be a cost-effective choice for managing ADHD in Iran when compared to MPH.

## Background

Attention deficit hyperactivity disorder (ADHD) is a neurodevelopmental disorder [1]. It is characterized by symptoms such as difficulty maintaining focus (inattention), excessive and inappropriate movement (hyperactivity), and impulsive behavior (acting without thinking) [2]. ADHD is a chronic and impactful disorder that significantly impacts various aspects of an individual’s life, including academic and professional achievements, relationships, and daily functioning [3]. When left untreated, ADHD can diminish self-esteem and social functioning, particularly in children [4]. Adults with ADHD may suffer from low self-worth, sensitivity to criticism, higher rates of singlehood, divorce, criminal behavior, and delinquency [5].

Approximately 8.4% of children and 2.5% of adults are estimated to have ADHD worldwide [6]. ADHD becomes apparent in school-aged children when it disrupts classroom settings or interferes with schoolwork [7]. Diagnoses are higher among boys, not because they are more prone to ADHD, but because symptoms manifest differently between genders [8]. Boys exhibit hyperactivity and other outward symptoms, while girls often display inattentiveness [9].

While many children may exhibit traits like restlessness, difficulty waiting their turn, inattention, fidgeting, and impulsivity, those who meet the diagnostic criteria for ADHD demonstrate significantly more pronounced symptoms in hyperactivity, impulsivity, organization, and inattention compared to what is expected for their age or developmental level [10]. These symptoms cause considerable distress and create challenges at home, school, work, and relationships [11]. It’s crucial to recognize that these symptoms are not due to defiance or an inability to understand tasks or instructions [12].

ADHD is categorized into three primary types: predominantly inattentive presentation, predominantly hyperactive/impulsive presentation, and combined presentation [13]. It has varying clinical characteristics, often accompanied by multiple comorbid conditions [14]. Frequently observed externalizing disorders that coexist with ADHD and make its diagnoses very challenging include oppositional defiant disorder (ODD) and conduct disorder (CD) [15]. The prevalent externalizing conditions among youngsters, including children and adolescents, encompass ADHD, CD, and ODD. These conditions are commonly labeled as disruptive behavior disorders (DBDs), given their shared trait of causing disturbances in environments such as home and school [15, 16]. ODD is marked by a consistent pattern of oppositional behaviors, including defiance in complying with parental, teacher, or other adult requests or instructions [15, 17]. The majority of these youngsters display resistance and rebelliousness towards authority figures, and they might engage in disruptive actions, yet they do not exhibit significant antisocial conduct [15]. Nevertheless, both boys and girls diagnosed with ODD face a heightened risk of developing more severe complications, primarily CD [15, 17]. CD constitutes an established pattern of oppositional and defiant behaviors, coupled with antisocial actions like theft, fighting, truancy, and intimidation [15, 17]. This disorder is classified into two forms: (a) early-onset CD, which emerges prior to the age of 7, and (b) late-onset CD, which surfaces in the preteen or early teenage years [15]. ADHD exhibits a tendency to co-occur with ODD and CD, although the connections between them vary [15, 17]. Among boys with ADHD, the likelihood of developing CD is greater compared to boys without ADHD [15]. This is primarily due to the high co-occurrence of ADHD and ODD. ODD seems to serve as a precursor to CD in boys, while ADHD does not have the same predictive role. Our understanding of these linkages in girls is considerably limited, as the associations might or might not mirror those observed in boys [15]. ADHD typically emerges during the preschool or early school years, and if coupled with early-onset CD, it results in more pronounced behavioral challenges throughout the elementary school phase [15].

Other diagnoses like disruptive mood dysregulation disorder (DMDD) and intermittent explosive disorder (IED) have also been observed to co-occur with ADHD [16]. Diagnosis is based on persistent symptoms that have occurred for at least 6 months and have been noticeable [17]. While ADHD can be diagnosed at any age, it typically begins during childhood [18]. To make a diagnosis, the symptoms must have been present before the individual turned 12 years old and must have caused difficulties in multiple settings, not solely at home [19].

The economic burden of untreated ADHD is substantial, as evidenced by the estimated cost of 12 billion dollars between 2018 and 2020 in the United States [20]. In Iran, studies suggest that approximately 5.0–7.0% of children are affected by ADHD [21]. However, a comprehensive analysis of the costs associated with this patient population and the overall financial burden on the country's healthcare system has yet to be conducted. Given the wide-ranging impact of ADHD, the disorder is likely to have significant economic consequences for affected children, families, and society [22]. Although research on financial costs is relatively recent, early studies suggest that ADHD increases healthcare expenditures and related costs [23].

Stimulant medications, such as Amphetamines (AMPH) and MPH, are widely recognized as effective first-line treatment options for most children and adults with ADHD [24]. Approximately 70.0% of patients respond favorably to first-line treatments [25]. However, it is essential to note that these approved medicine classes for ADHD treatment in European and North American countries are not without side effects [26]. AMPHs are non-catecholamine sympathomimetic amines that stimulate the central nervous system (CNS) [27]. They inhibit the reabsorption of norepinephrine and dopamine into the presynaptic neuron while increasing their release in the intraneuronal space [28]. LDX is an oral osmotic release medication known for its therapeutic efficacy, particularly in children under 18 [29]. LDX, a prodrug of Dextroamphetamine, is utilized in the treatment of ADHD in both children and adults [30]. In Iran, it is marketed under the brand name Vyas [31]. Alongside stimulant medicines, non-stimulant medications serve as alternative treatment options for ADHD patients [32]. Atomoxetine (ATX), a non-stimulant medicine, has been approved by the Medicines and Healthcare Products Regulatory Agency (MHRA) in England for adult ADHD patients presenting with childhood-onset symptoms [33]. The National Institute for Health and Care Excellence (NICE) has recently recommended LDX for inclusion in the first-line list of ADHD medications [34]. Should patients prove resistant to LDX and MPH, among the very limited list of medicines available in the Iranian pharmaceutical market, ATX can serve as a suitable second-line alternative [35].

This study aims to estimate the cost-effectiveness and conduct a BIA of LDX versus MPH as a first-line treatment for ADHD. CEA helps us choose the best disease treatment strategy [36]. Using more cost-effective medications can significantly reduce the long-term costs associated with treating this disorder, as demonstrated by the BIA over five years. By utilizing decision-analytic modeling, this analysis provides evidence-based information for policymakers in Iran and other Developing Middle Eastern Countries (DMECs), facilitating the efficient allocation of healthcare resources.

## Method

**Fig. 1:**
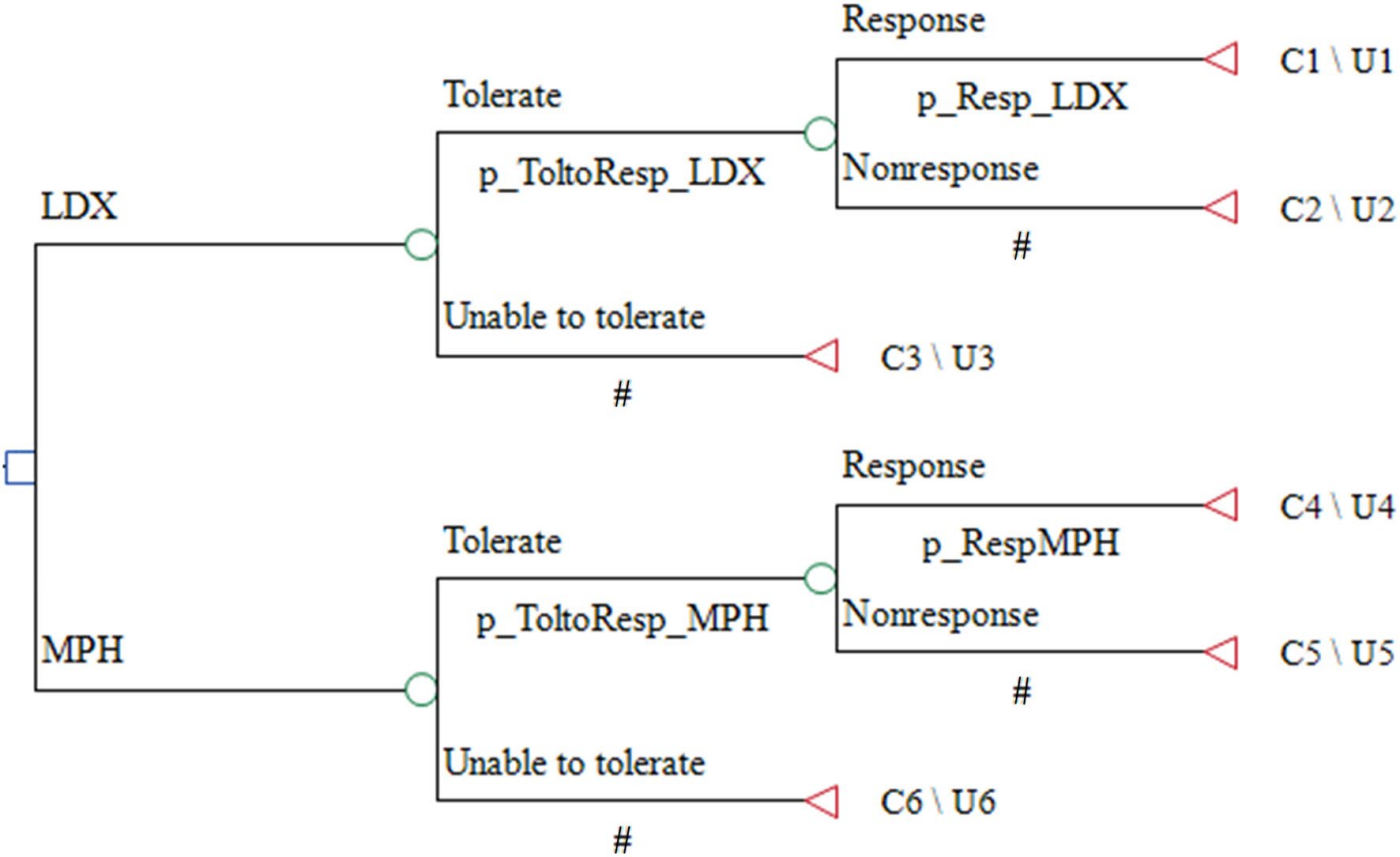
Model structure as a decision tree model in Treeage pro healthcare 2022 for ADHD in patients under 18 years old in Iran

Base Case Study Design: the study employed a CEA to compare the effectiveness and reduction in direct and indirect costs per case of ADHD when treated with LDX and MPH from a social perspective. A one-year decision-analytic modeling approach was used to assess the value for money and financial consequences of the new health intervention (Fig. 1).

### Model inputs

#### Clinical data

The target population for this research consisted of Iranian children and adolescents under 18 years old diagnosed with ADHD by a psychiatrist or neurologist based on DSM-5 guidelines. The study monitored patients who visited the psychiatric hospital in Mashhad for examination by Psychiatrists or neurologists and those who obtained ADHD medications from governmental pharmacies over 3 months. We included all the patients who had the above conditions in the study, and the requirements for excluding them from the analysis were their lack of consent to continue participating in the survey or stop taking the medicine. Eventually, a total of 197 individuals were included in the study—134 from Mashhad Psychiatric Hospital and 63 patients from governmental pharmacies. It should be mentioned that preschool patients mostly resisted behavioral therapies, and according to the guidelines, in this situation, psychiatrists started pharmacological treatments for them [19, 26]. Clinical data were collected through interviews conducted by neurologists and psychiatrists. Demographic information about the patients can be found in Table 1. During the interviews, EQ-5D questionnaires, which is one of the valid and standard questionnaires to evaluate the quality of life, were completed by patients and their parents, providing valuable clinical data. Some of these patients, who were over 7 years old, independently completed this questionnaire. The rest, who were under 7 years old and lacked the ability to read and write, or due to physical and mental conditions, were not in the circumstances to complete the questionnaire alone, had this questionnaire completed with the assistance of their parents. Additionally, the study considered the findings of relevant studies and trials conducted in other countries that demonstrated the effectiveness of LDX and MPH in treating ADHD [37–41]. These studies consistently showed the superiority of medicine-based treatment over placebo [41, 42]. EQ-5D questionnaires were utilized to validate the results of these studies and trials. Thus, the clinical data necessary for the CEA were collected. The target population for this research consisted of Iranian children and adolescents under 18 years old diagnosed with ADHD by a psychiatrist. The baseline transition probabilities for treatment response and discontinuation due to adverse events were obtained from the Zimovetz study, as presented in Table 2 [39].

**Table 1:** Frequency of some demographic characteristics (based on sex) of patients participating in the study

Demographic characteristics		Male number (percentage)	Female number (percentage)
		110 (55.83)	87 (44.16)
Age	Under 5 years	38 (34.54)	22 (25.28)
	5–12 years	53 (48.18)	46 (52.87)
	12–18 years	19 (17.27)	19 (21.83)
Place of residence of the patient in terms of urban/rural	Urban	85 (77.27)	59 (67.81)
	Rural	25 (22.72)	28 (32.18)
Education	Not school aged	39 (35.45)	27 (31.03)
	Primary	44 (40.0)	34 (39.08)
	High school	27 (24.54)	26 (29.88)
Having insurance	Yes	89 (80.90)	69 (79.31)
	No	21 (19.09)	18 (20.68)
Insurance type	No insurance	21 (19.09)	18 (20.68)
	Iranian health insurance	53 (48.18)	43 (49.42)
	Social security insurance	28 (25.45)	22 (25.28)
	Armed forces insurance	8 (7.27)	4 (4.59)
Supplementary insurance	Yes	28 (25.45)	24 (27.58)
	No	82 (74.54)	63 (72.41)

**Table 2:** Relative risks for discontinuation due to adverse events (medicine vs. placebo)

Treatment	Relative risk (95.0% CI)	Placebo risk (95.0% CI)
LDX	3.21 (0.93–7.90)	0.0443 (0.035–0.053)
ATX	2.67 (1.68–4.13)	
MPH-ER	2.76 (1.83–4.07)	

*LDX* Lisdexamfetamine, *ATX* Atomoxetine, *MPH-ER* Methylphenidate extended release, *CI* confidence interval

### Costs

#### Direct medical costs

The study considered direct medical costs, specifically medicine acquisition costs. Unit costs were obtained from the official FDA website of Iran in June 2020 and converted to 2022 US Dollars (USD), with an exchange rate of 42,000 Iranian Rial Rates (IRR) per USD [43]. To account for potential economic fluctuations and exchange rate changes, the study used the free-market exchange rate obtained from the foreign exchange market in Iran for the statistical calculations of direct and indirect costs. The conversion rate used was 290,427 Rials per US Dollar, announced by the official exchange offices of the Central Bank of Iran at the time of the research.

The trials and studies used 30- and 50 mg dosage forms of LDX [37–41]. Therefore, the average price of both forms was considered. This approach was justified by the LDX Defined Daily Dose (DDD), which is 30 mg daily [44]. Regarding MPH, the trials and studies utilized a daily dose ranging from 10 to 60 mg, but the study adopted the WHO-recommended amount of 30 mg [45]. As Iran is not a member of the World Trade Organization (WTO), it does not strictly adhere to intellectual property laws for pharmaceutical patents. Consequently, pharmaceutical products in Iran are available in Original Brand (OB), Generics (Gx), and Biosimilar (BS) forms. The unit prices for general forms of MPH were 0.004US Dollars per 1 mg, while Gx forms of LDX long-acting capsules were the only available forms in Iran, with unit prices of 0.013US Dollars per 1 mg.

All costs were calculated in 2022 US Dollars, with an exchange rate of USD 1 = IRR 290,427 (Iranian Rial). The study utilized a cost-effectiveness threshold of 2450 USD, as the WHO recommended, based on the latest acceptable CEA threshold announced by the Iran FDA (Table 3).

**Table 3:** Direct and indirect costs applied in the base-case

Resource item		Lisdexamfetamine ($)	Methylphenidate ($)
Direct medical cost	Psychiatrist	23.55 (12.40–49.58)	34.43 (12.40–49.58)
	Psychologist	17.22 (0–84.70)	34.69 (17.21–43.04)
	Supplementary medicines	11.88 (6.20–24.79)	41.32 (4.13–123.96)
	Medical tests	15.49 (1.03–41.32)	15.49 (1.03–41.32)
	The total cost of outpatient services	6.89 (0–189.38)	6.89 (0–117.07)
	The cost of the leading medicine	252.44 (139.88–365.00)	19.37 (8.89–40.00)
Direct non-medical cost	Intra-city transportation	9.74 (6.20–22.38)	19.63 (6.20–33.05)
	Travel to and accommodation in the treatment city	110.18 (30.99–137.73)	84.36 (30.99–103.30)
Indirect cost	Salary of nurse/caregiver	17.22 (0–206.5)	8.61 (0–219.68)
Total patient costs (per 28 days)		464.61 (196.70–2979.88)	264.79 (80.85–434.25)

#### Direct nonmedical costs

Direct nonmedical costs were gathered through self-declaration by patients with ADHD. Face-to-face or telephone interviews were conducted using a pre-prepared checklist to calculate these costs. Direct nonmedical prices include expenses related to transportation (within the city and long-distance), food and accommodation for the patient and their companions, purchase of medical supplies and aids (such as wheelchairs, walkers, and home care beds), home modifications due to the illness (e.g., installation of an elevator for a paralyzed patient with ADHD), and the cost of accommodation for patients' companions (Table 3).

#### Indirect costs

Indirect costs were derived from the productivity loss of patients or their family members due to illness, death, or treatment. The productivity loss includes the absence from the work of patients and their family members who provide care. The following factors were calculated as productivity losses in this study:

- Number of disability days for patients and companions, including time spent on outpatient and inpatient services, travel time, hospitalization days, and recovery days after discharge.
- Job loss resulting from illness.
- Number of hospitalizations, nursing days at home, and days of disability for family members, relatives, and friends due to patient care.
- Percentage decrease in patient income due to illness.

The study employed the human capital approach based on the minimum wage to calculate indirect costs. The data necessary for this calculation were obtained through self-reporting by patients and their companions via face-to-face or telephone interviews.

The formula used to calculate the indirect cost for each individual and disease status is as follows: Minimum daily wage * Total number of disability days for patients and companions = Indirect cost.

The average national wage for laborers in Iran was determined to be 885,165 Iranian Rials (USD 1 = IRR 279,199) per day, multiplied by the number of days lost [46]. The minimum monthly wage was 41,797,500 Rials (USD 149.70), and the minimum daily wage was 1,390,000 Rials (USD 4.97) [46].

#### Model assumptions

Patients are included in the model when they start treatment with either LDX or MPH. They undergo a 28-day titration period to reach the optimal treatment dose. Patients who experience intolerable side effects during titration (within the first 14 days of treatment) discontinue therapy. For those who stop treatment during titration, the utilities and costs during this period (28 days) are a combination of 50% respondent and non-respondent values. The costs are also a mixture of 50% respondent costs and 50% non-respondent medicine costs. This assumption is based on the average observation that patients discontinue treatment halfway through the first month across different treatments. Alternative assumptions were explored where these patients were assumed to have the same utility as respondents and non-respondents during titration.

Patients who discontinue treatment due to intolerable side effects do not start any further pharmacological treatment. Like non-respondents, they are assumed to receive behavioral therapy. This assumption is primarily driven by the need for more relevant clinical evidence on follow-up therapies. In the model, the behavioral therapies are the same for both LDX and the comparison group, so the model results do not differentiate between them. Therefore, it is assumed that patients who drop out have the same utilities and costs as non-respondents for the remaining 1-year model horizon.

At the end of the titration period, patients who do not respond to treatment discontinue it without pursuing other pharmacological therapies. They are assigned the non-response costs and utilities for the titration period and the remaining time horizon of the model. Patients who respond to treatment at the end of the titration period continue with it for the rest of the model's time horizon, maintaining their level of response. Patients who respond to and tolerate treatment are assumed to be fully adherent and persistent throughout the model's time horizon, based on observations from pivotal trials. This dichotomous response framework is utilized. In the base-case evaluation, costs and outcomes are not discounted since the time horizon is 1 year.

#### Treatment strategies

In the medical approach for ADHD patients, two pharmacological treatment options were considered: LDX and MPH. The initial dose for LDX was 30 mg, followed by a maintenance dose of 30 to 70 mg once daily [47]. For MPH, the initial amount was 5 mg twice daily, which could be adjusted to a maximum of 60 mg daily [48]. The medicines were intended to be used for 1 year only.

#### Model outputs

The study’s primary outcome was the ICER. This ratio represents the additional cost of pharmacological treatment to achieve a designated clinical outcome (QALY) within 1 year. The effectiveness and cost-effectiveness of interventions were calculated and compared using monetary units, QALYs, and the cost per QALY for all treatments. The ICER was estimated using the formula [36]: ICER = (Cost of intervention A—Cost of intervention B)/(Increase in quality-of-life A—Increase in quality-of-life B).

#### Scenario analysis

The decision tree model used in the study considered two treatment strategies for patients receiving LDX and MPH. In each strategy, patients could either tolerate the medications or discontinue treatment. After the titration stage, patients had two conditions: they either responded to treatment or did not respond. Clinical guidelines such as NICE, the American Academy of Pediatrics (AAP), and the European ADHD Guidelines Group (EAGG) and expert opinions guided the selection of treatment comparators [49–51]. Non-pharmacological interventions, such as parent training, cognitive behavioral therapy (CBT), cognitive training, play therapy, and Biofeedback and Neurofeedback, were included as part of the non-medicine costs and were assumed to vary based on treatment response [52]. The target population for the analysis was children and adolescents with ADHD. Health outcomes included "tolerate," "unable to tolerate," "response," and "non-response." The impact of using LDX instead of MPH in terms of costs and health outcomes was assessed based on treatment response and discontinuation rates.

#### Sensitivity analysis

Deterministic and probabilistic sensitivity analyses were conducted to address uncertainties in model inputs. In DSA, ± 20.0% variation in necessary information such as average cost of ADHD care, prices of LDX and MPH, and 5.0% variation in the probability of ADHD in the first year were considered. PSA was utilized using Monte Carlo simulation with 5000 iterations to generate a scatter plot and acceptability curve, capturing the uncertainties in the model.

#### Budget impact analysis

The DDD of LDX and MPH were obtained from the WHO website for the budget impact analysis. The number of people with ADHD and the prevalence rate among individuals under 18 years old in Iran was considered. Market share calculations were performed using official pharmaceutical statistics from the Food and Medicine Organization of the Ministry of Health in Iran. Three scenarios were designed to calculate the budget impact of adding LDX:

1. LDX is not covered by insurance, and treatment continues with MPH.
2. LDX is not covered by insurance, but the medicine is available in the market.
3. LDX is covered by insurance, and both LDX and MPH are used for treatment, considering changes in market share. The market size of MPH and other competitors was estimated.

## Results

Table 1 presents an overview of the demographic characteristics of each diagnostic group, encompassing the reviewed patient’s age, place of residence of the patient, education, and being covered by insurance. The majority of participants in both the boy’s and girl’s groups were aged between 5 and 12 years, accounting for 52.87% and 48.18%, respectively.

The decision tree model underwent a year-long process. As part of this study, it was assumed that patients were prescribed either LDX or MPH. The transition probabilities for cost-effectiveness comparison were obtained from another study using the LDX/MPH tolerate/unable to tolerate coefficient [39]. By applying these coefficients to the baseline transition probability matrix, the transition probability matrix for patients receiving LDX/MPH was calculated (Table 2).

### Base-case analysis results

Table 4 displays the cost-effectiveness results obtained from the decision tree estimation model. To calculate the cost of each diagnostic method, the associated probabilities for each branch are multiplied by the specific cost of that branch. Based on the findings, it became clear that utilizing LDX was a dominant strategy compared to MPH. This conclusion was supported by the fact that LDX not only demonstrated higher effectiveness (0.208) but also incurred higher costs ($54.9). The total expenses per patient for LDX and MPH over a 1-year duration were $750 and $695, respectively. Furthermore, the total 1-year QALY, with a maximum possible value of 1, was determined to be 0.765 for LDX and 0.557 for MPH.

**Table 4:** Base-case analysis results (per patient)

Strategy name	Total costs ($)	Total QALYs	ICER (Cost per QALY)	Incremental costs ($)	Incremental QALYs
MPH	695	0.557	0	0	0
LDX	750	0.765	264.28	54.9	0.208

*LDX* Lisdexamfetamine, *MPH* Methylphenidate

The findings revealed that the use of LDX therapy could lead to a reduction in expenses related to psychiatrists and psychologists. Consequently, this could prevent potential losses in QoL and associated costs. The analysis of cost elements demonstrated that travel and accommodation in the treatment city constituted the highest percentage of total treatment costs for both options for curing ADHD (Table 3).

### Sensitivity analysis results

#### Deterministic sensitivity analysis

**Fig. 2:**
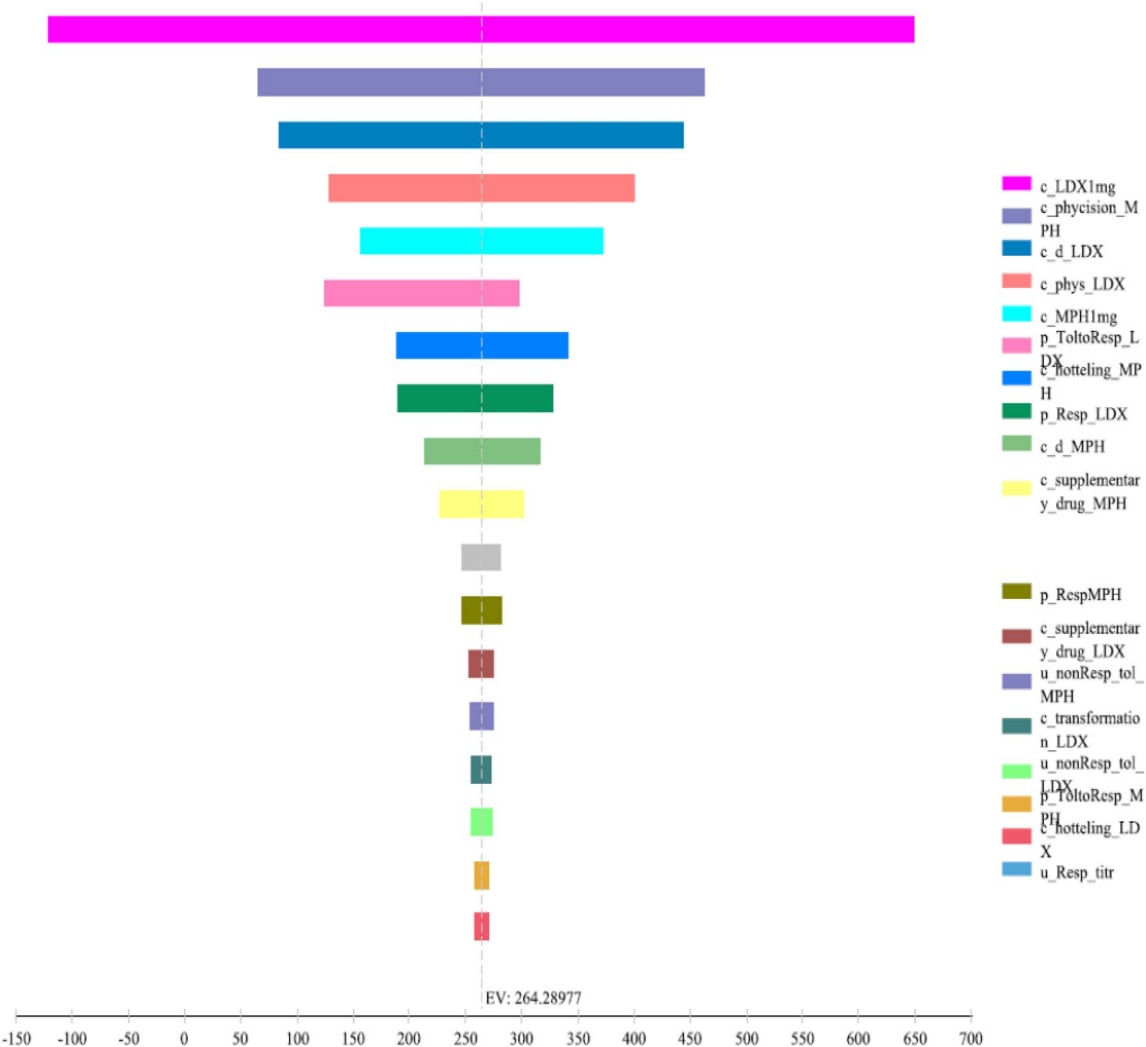
Results for one-way sensitivity analysis, effect of parameter variation on the incremental cost (USD) per QALYs (Tornado test)

The results of the DSA are presented in the Tornado Diagram (Fig. 2). These findings demonstrate that the ICER is inclined to remain below the threshold of USD $2450 per QALY, as indicated by all analyses. Furthermore, the results indicate robustness to clinically acceptable changes in assumptions, including a cost range of 20% and a variability range of 10% for costs and utilities, respectively.

#### Probabilistic sensitivity analysis

**Fig. 3:**
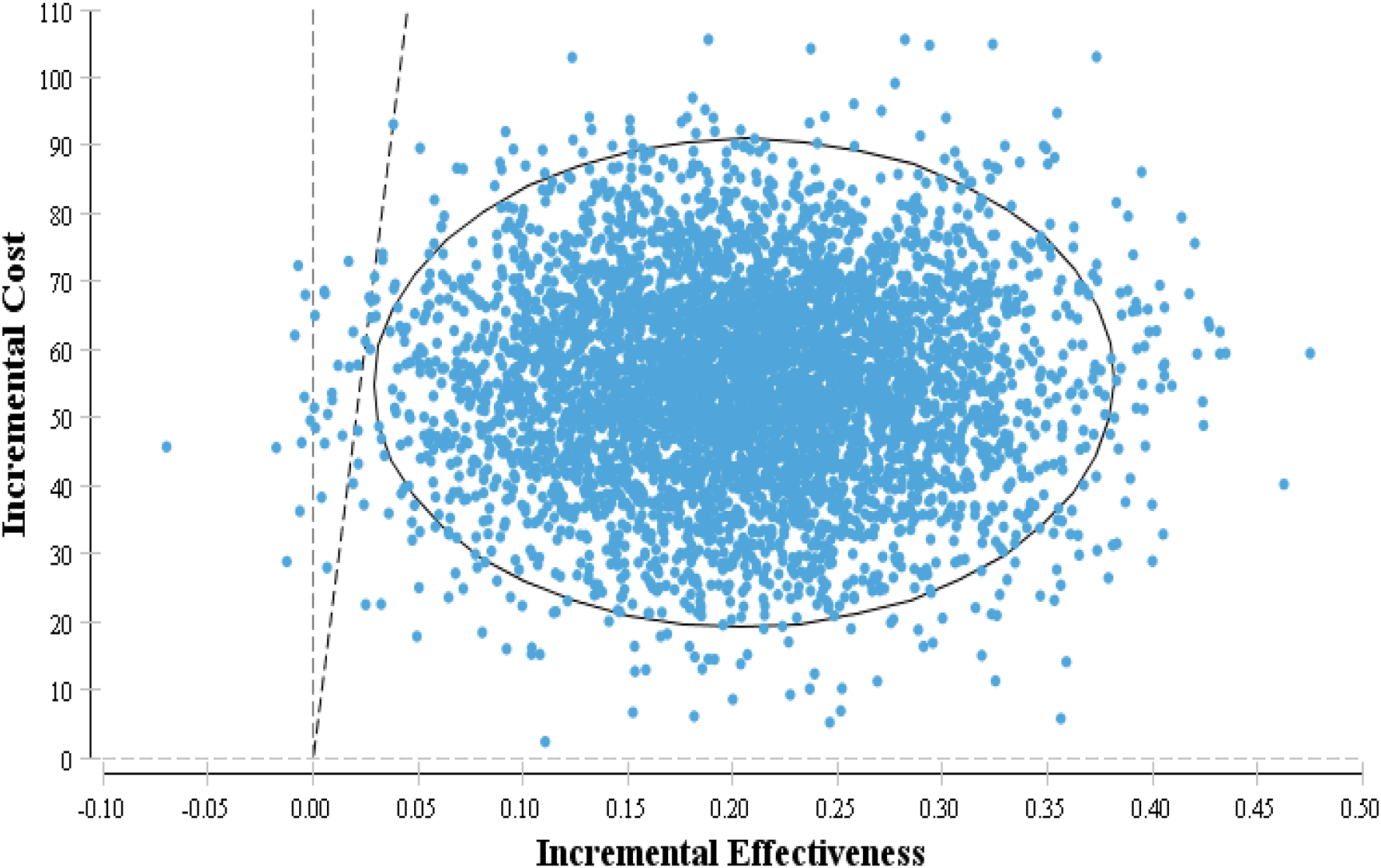
Incremental cost-effectiveness scatter plot of LDX vs. MPH in patients under 18 years old in Iran

In PSA, the parameters are defined as a distribution rather than a singular point. The results of the PSA indicated that LDX has a 0.994 percent probability of being cost-effective when compared to MPH, using a threshold of USD 2450 (Fig. 3).

**Fig. 4:**
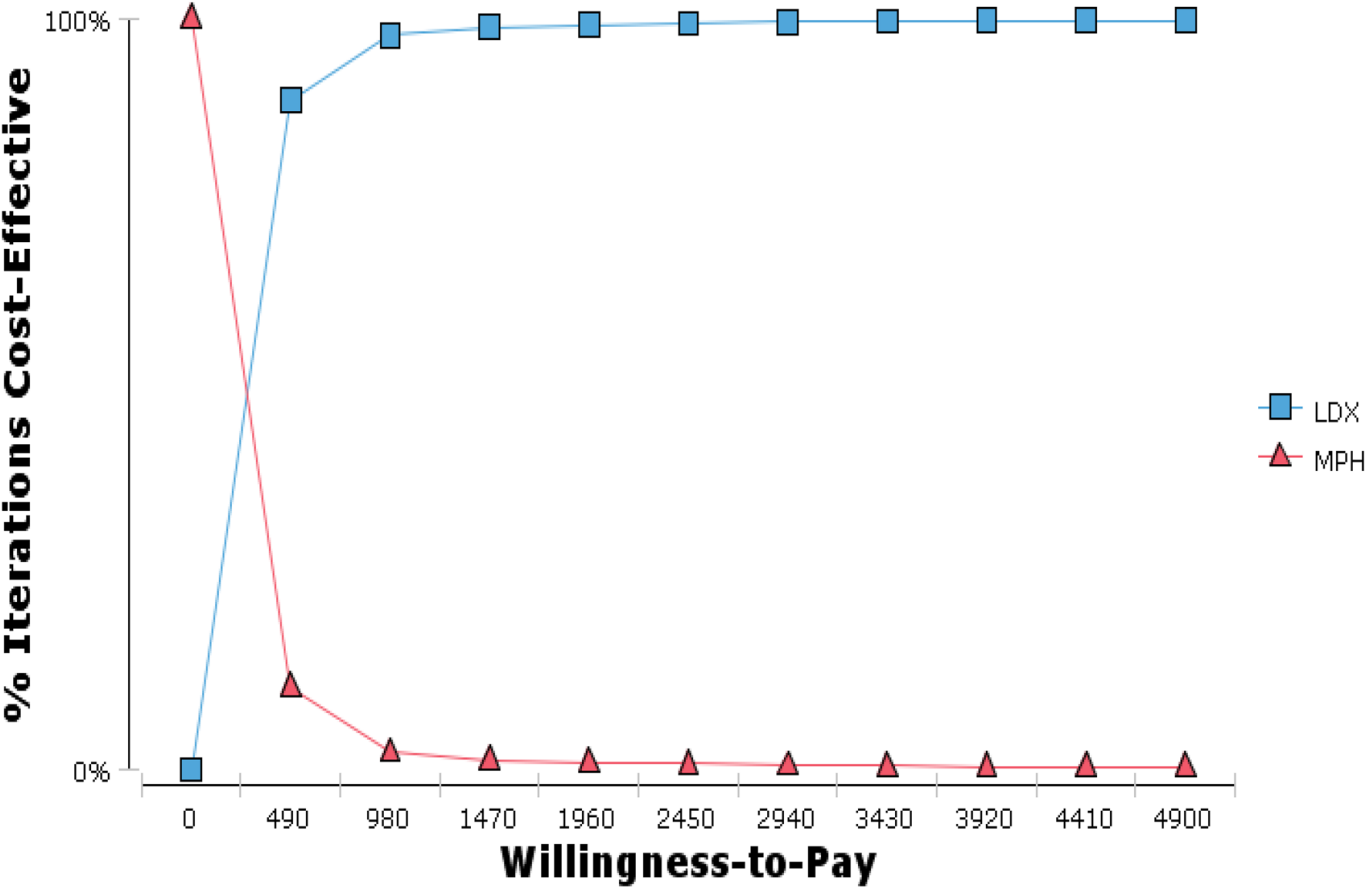
Cost-effectiveness accessibility curve of LDX vs. MPH for estimating the willingness to pay for LDX in treating ADHD for patients under 18 years old in Iran (Monte Carlo simulation)

The cost-effectiveness acceptability curve demonstrated that, at a threshold exceeding USD 400, LDX becomes a cost-effective treatment in Iran (Fig. 4). Table 5 provides a summary of the variables utilized in the DSA and PSA.

**Table 5:** Model parameter

Parameters	Base-case value	Range	Distribution	References
Age of patient	Under 18 years	Fixed	Fixed	
Cost discount rate per annum	7.2%	3.0–10.0%	Fixed	[59]
Effects discount rate per annum	5.0%	3.0–10.0%	Fixed	[60]
DSA				
Probability of tolerance in MPH	0.936	0.842–1	Fixed	
Probability of response in LDX	0.882	0.792–0.968	Fixed	
Probability of response in MPH	0.841	0.756–0.924	Fixed	
Probability of tolerance in LDX	0.959	0.863–1	Fixed	
Utility of response in titration	0.381	0.342–0.418	Fixed	
Cost of LDX per 1mg ($)	0.013	0.010–0.015	Fixed	
Cost of MPH per 1mg ($)	0.004	0.003–0.005	Fixed	
Cost of the physician in LDX ($)	34.43	27.54–41.32	Fixed	
Cost of hoteling in MPH ($)	84.36	67.48–101.24	Fixed	
Cost of hoteling in LDX ($)	6.89	5.51–8.26	Fixed	
Cost of the supplementary medicine in LDX ($)	11.88	9.50–14.25	Fixed	
Cost of the supplementary medicine in MPH ($)	41.32	33.05–49.58	Fixed	
Cost of transformation in LDX ($)	9.74	7.79–11.68	Fixed	
Cost of transformation in MPH ($)	19.63	15.70–23.55	Fixed	
The utility of nonresponse is tolerated in LDX	0.68	0.612–0.768	Fixed	
Utility of nonresponse in tolerate in MPH	0.59	0.53–0.65	Fixed	
PSA				
Utilities				
	mean	SD	Distribution	
Utility in response to titration in MPH	0.38	0.02	Normal	
Utility of response in treatment in LDX	0.85	0.08	Normal	
Utility in non-response in titration	0.68	0.03	Normal	
The utility of nonresponse is tolerated in LDX	0.68	0.03	Normal	
Utility of response in treatment in MPH	0.61	0.06	Normal	
Utility of nonresponse in tolerating MPH	0.59	0.05	Normal	
Transitional probabilities used in the 1-year decision tree				
Probability in response to MPH group	0.84	0.04	Beta	[39]
Probability in response LDX group	0.88	0.05	Beta	
Probability of tolerating response in the LDX group	0.96	0.05	Beta	
Probability of tolerating response in the MPH group	0.94	0.05	Beta	
Cost distribution of LDX ($)	728	110	Gamma	
Cost distribution of MPH ($)	886	113	Gamma	
LDX daily dose	51.52	2.62	Normal	
LDX daily dose in titration	25	1.67	Normal	
MPH daily dose	50.93	2.59	Normal	

*LDX* Lisdexamfetamine, *MPH *Methylphenidate, *mg* milligram, *DSA* Deterministic Sensitivity Analysis, *PSA* Probabilistic Sensitivity Analysis, *$* United States Dollars

The PSA indicated that the model was robust based on the distribution of parameters such as clinical efficacy, costs, and utilities. In addition, the PSA demonstrated that LDX was the optimal strategy as it was cost-effective in more than 99.4 percent of simulations at a Willingness to Pay (WTP) threshold of 1 GDP per capita.

#### Budget impact analysis

**Fig. 5:**
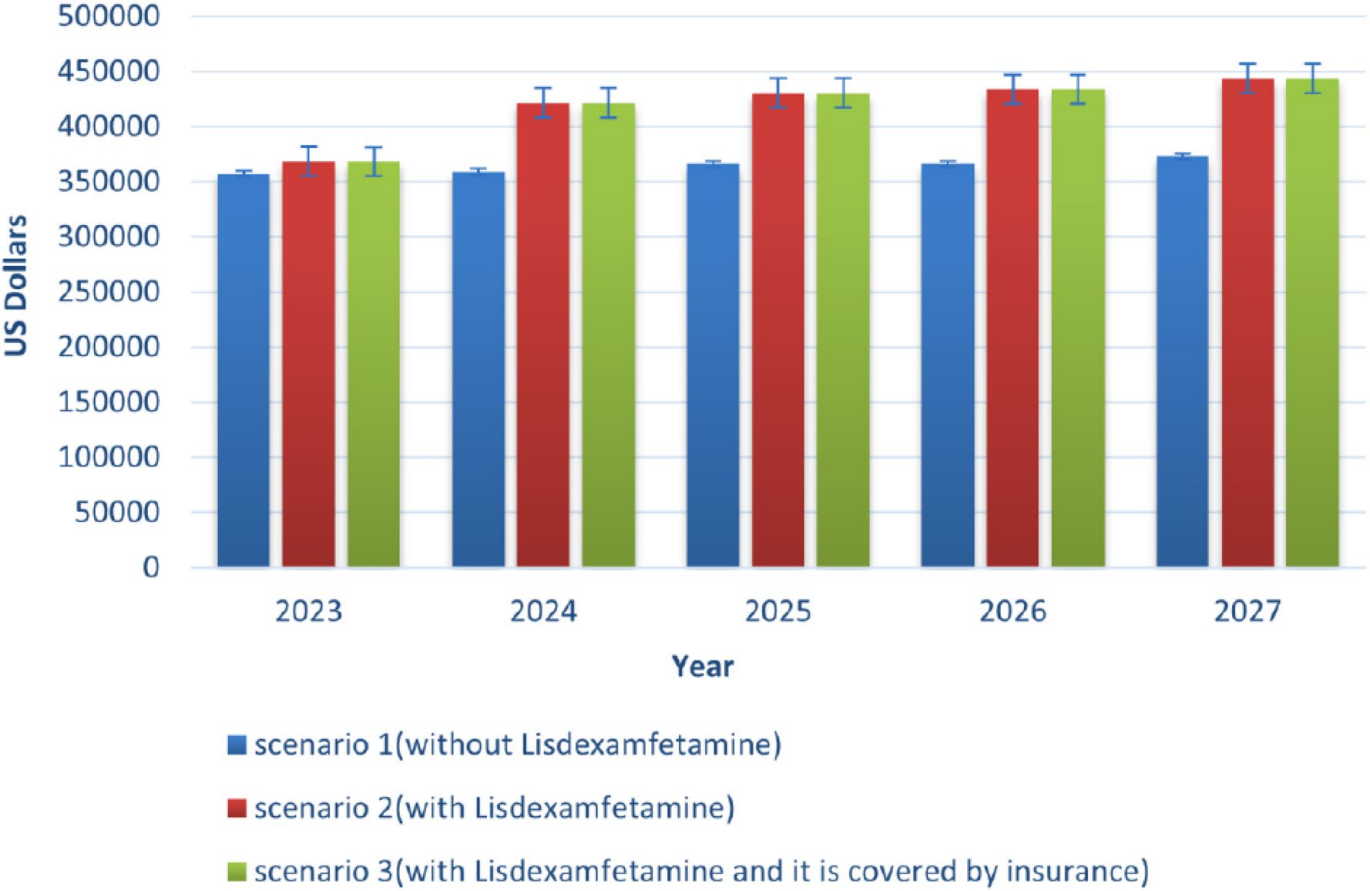
comparison of three different scenarios in budget impact analysis

The information utilized to estimate LDX costs is depicted in Table 6. The total cost of treating patients was determined by considering the estimated number of patients and the cost of treatment per patient. The findings indicate that the inclusion of LDX in Iran's drug list does not significantly burden the healthcare system in terms of expenses. In the initial year (2023), the projected cost of treating ADHD without LDX was approximately $357,412. However, with the addition of LDX, the cost increased to about $368,566. This trend continued in subsequent years. It is worth noting that while incorporating LDX into Iran's drug list will lead to increased costs for the country and society, it will also enhance patient treatment and quality of life. Over a period of five years (2023–2027) of LDX usage, the incremental cost amounts to $75,313, starting from $368,566 in the first year and reaching $443,879 in the fifth year (Fig. 5).

**Table 6:** BIA analysis results

Year	2023	2024	2025	2026	2027
Iran population	89613176.84	90688534.96	91776797.38	92878118.95	93992656.38
ADHD patients	13979655.59	14147411.45	14317180.39	14488986.56	14662854.39
Market share of methylphenidate	0.27	0.12	0.12	0.10	0.09
Market share of atomoxetine	0.35	0.44	0.43	0.46	0.46
Market share of clonidine	0.39	0.44	0.45	0.44	0.46
Market share of lisdexamfetamine	0.01	0.02	0.03	0.04	0.05
Market share of lisdexamfetamine (IF it is covered by insurance)	0.01	0.03	0.05	0.08	0.11
Scenario 1(without Lisdexamfetamine) ($)	357412	359048	365994	366395	372724
Scenario 2 (with lisdexamfetamine) ($)	368566	421487	430407	433828	443879
Scenario 3 (with lisdexamfetamine and it is covered by insurance) ($)	368566	421483	430,397	433808	443849
Financial impact in scenarios 1&2 ($)	11154	62439	64413	67432	71154
Financial impact in scenarios 1&3 ($)	11154	62434	64403	67412	71124

*ADHD* Attention-Deficit/Hyperactivity Disorder, *$* United States Dollars

## Discussion

We estimated the disease management cost for ADHD through micro-costing measurement, filling the relevant research gap in Iran; the investigation results show that the cost rises with the severity of the disease. Thus, treating the disease as early as possible is essential to postpone its progression [20, 21]. The result of this economic evaluation suggested that LDX is costly and adding it to the treatment protocols of ADHD patients would have a financial burden on the economic part of Iran’s health system; it was a cost-effective strategy as compared with MPH for the treatment of ADHD patients under 18 years old diagnosed with ADHD by neurologists and psychiatrists in Iran. In the current study, a decision tree method was used to assess the cost-effectiveness of LDX compared with MPH in a 1-year horizon for ADHD patients in Iran from a society’s perspective. The results indicated that adding LDX to the medicine regimen can significantly reduce the complications and problems of the disease and greatly increase the quality of life of patients with this disease.

The cost of LDX accounts for approximately 50% of the total treatment cost. The difference in the impact on health insurance between considering LDX medicine cost and the overall treatment is slight. In addition, because of the high price of LDX, the expected number of patients who use LDX is based on the ability to pay rather than the other factors.

The findings of our study were consistent with similar studies on LDX and Methylphenidate in various countries [34, 37, 52–55]. Generally, the economic evaluations revealed that, although LDX was costly, its significant reduction in the symptoms and complications of the patients made it cost-effective over MPH, with its ICER falling below the conventional threshold values.

DSA was undertaken to reflect how robust the results were to the model parameters and assumptions, and it found that the findings remained relatively the same in most scenarios. As noted, the model was the most sensitive to acquisition costs for LDX. The scatter plot generated by the probabilistic sensitivity analysis (PSA) indicated that LDX had a higher acceptance rate in most cases than MPH. Specifically, LDX fell within the acceptance area and below the threshold in 99.4% of cases, making it a more cost-effective strategy than MPH. Additionally, the cost-effectiveness acceptability curve derived from the PSA revealed that LDX was the most cost-effective treatment when the threshold was set below $2450.

BIA is an economic evaluation applied to assess the variations in spending of a definite budget holder if a different health technology/program is used [56]. A BIA helps policy and decision-makers in health care service planners and officers decide if a program or intervention is affordable according to resource scarcity and budget constraints. In contrast, an economic evaluation such as cost-effectiveness and cost-utility analysis notifies decision-makers about whether a program or intervention is good value for money [57]. This study included models for assessing the budget impact of LDX for treating ADHD over 5 years. According to the International Society for Pharmacoeconomics and Outcomes Research (ISPOR), we used a time horizon for BIA [58]. The results of this analysis indicate that according to scenarios one and two, the budget impact would increase by year from 2023 to 2027. This analytical approach helps to better decision-making by policymakers in middle and low-income countries about the best resource allocation in their health program planning.

To the best of our knowledge, this study is the first economic evaluation of the cost-effectiveness and budget impact of LDX for treating ADHD patients in Iran. Ministry of Health, health insurance organizations, and national medicine regulatory agencies in Iran and other middle-income countries can use the results of this study for policymaking in developing and implementing clinical guidelines, pricing, and reimbursement of LDX in ADHD. Economic evaluations of medicines in low- or middle-income countries are uncommonly performed. More economic evaluations, such as the one presented, are required to assess the cost-effectiveness of new medicines in such countries for guiding policy decision-making.

So far, limited studies have been conducted to investigate medicine treatments in patients with ADHD. Still, in most studies, the following essential aspects regarding cost calculations have yet to be considered. In most published studies, only direct costs are considered, and indirect costs are not included despite their high importance. Also, quitting medicine therapy and medicine switching are not considered [39, 55].

The cases mentioned above are among the weaknesses of previously published studies, and in this study, several limitations applied in previous studies have been removed. Also, as it was said, because no similar research has been done inside the country and the WHO Eastern Mediterranean Regional Office (EMRO) in the field of the subject, and on the other hand, due to the prevalence of approximately 8% in the world for the mentioned disease and because of the lack of much information about the situation of this disease in the country, the implementation of this research can be of particular importance.

Among the limitations of this study regarding the disease prevalence information, such as the exact number of ADHD patients receiving treatment in different situations in the country, documentary evidence was unavailable. It should be noted that this problem is not specific to this disease. In most conditions, it is observed in terms of weakness in the data registration structure in Iran.

Time process to establish coordination for access to doctors and especially patients. Following up on patients is difficult in Iran due to the need for more public health surveillance systems. People's inherent abilities to respond to therapeutic and rehabilitative care differ, which can affect the research results, which is out of the researcher's control. Most of the patients in this study were children and teenagers. The ability of these people to answer the questions of the cost and quality of life questionnaires and the difference in the attitude of the patient’s parents and themselves towards increasing the quality of life after taking the medicine was considered a severe challenge to the researcher. Psychological support and care by family members are entirely individual and different for each patient, and this can affect the research results, which is beyond the researcher's control. One of the potential limitations is the possible oversimplification of complicated clinical outcomes. The other limitation is that the payers’ perspective does not capture proper healthcare and patient direct and indirect costs. Hence, a complete analysis from a broader (societal) view may be worthwhile. Although the time frame looks adequately long, a longer time frame, such as a lifetime horizon, will probably affect results. However, long-term inputs are lacking to inform a longer time horizon. We caution the reader that the generalizability of this study results in other healthcare settings may be limited. The cost differences between middle-income countries (such as Iran) versus high-income countries should be considered.

## Conclusion

The findings of this study support the inclusion of LDX as a treatment option for ADHD patients under 18 years old in Iran. Despite the higher cost compared to MPH, the significant reduction in symptoms and complications and improved quality of life make LDX a cost-effective choice. DSA and PSA demonstrated the robustness of the results, and LDX was consistently favored as the optimal strategy. In summary, this study contributes to understanding the economic evaluation and budget impact of LDX for ADHD treatment in Iran. It provides valuable insights into the cost-effectiveness of LDX and emphasizes the importance of considering both clinical outcomes and financial implications when making treatment decisions for ADHD patients.

## Data Availability

The datasets used and analyzed during the current study are available from the corresponding author upon reasonable request.
